# An Electrochemical Immunosensor Based on Chitosan–Graphene Nanosheets for Aflatoxin B1 Detection in Corn

**DOI:** 10.3390/molecules29071461

**Published:** 2024-03-25

**Authors:** Shuai Zhang, Caizhang Wu, Zhike Zhao, Kun Xu

**Affiliations:** 1 Key Laboratory of Grain Information Processing and Control (Henan University of Technology), Ministry of Education, Zhengzhou 450001, China; z.shuai69@stu.haut.edu.cn; 2 Henan Key Laboratory of Grain Photoelectric Detection and Control, Henan University of Technology, Zhengzhou 450001, China; 3 College of Electrical Engineering, Henan University of Technology, Zhengzhou 450001, China; zhaozhike@haut.edu.cn

**Keywords:** electrochemical immunosensor, aflatoxin B_1_, chitosan, graphene nanosheets, nanocomposites

## Abstract

We reported a highly efficient electrochemical immunosensor utilizing chitosan–graphene nanosheets (CS-GNs) nanocomposites for the detection of aflatoxin B_1_ (AFB_1_) in corn samples. The CS-GNs nanocomposites, serving as a modifying layer, provide a significant specific surface area and biocompatibility, thereby enhancing both the electron transfer rate and the efficiency of antibody immobilization. The electrochemical characterization was conducted utilizing both differential pulse voltammetry (DPV) and electrochemical impedance spectroscopy (EIS). Moreover, the antibody concentration, pH, antibody immobilization time, and immunoreaction time, were optimized. The results showed that the current change (ΔI) before and after the immunoreaction demonstrated a strong linear relationship ($R^{2}=0.990$) with the AFB_1_ concentration, as well as good specificity and stability. The linear range extended from 0.05 to 25 ng/mL, with a detection limit of 0.021 ng/mL (S/N=3). The immunosensor exhibited a recovery rate ranging from 97.3% to 101.4% in corn samples, showing a promising performance using an efficient method, and indicating a remarkable prospect for the detection of fungal toxins in grains.

## 1. Introduction

Aflatoxin is a highly toxic furanocoumarin derivative produced by Aspergillus flavus and Aspergillus parasiticus. It is frequently encountered in moldy grains such as rice, soybeans, and peanuts [1]. Aflatoxin B_1_ (AFB_1_) is known for its extreme toxicity and is widely acknowledged as one of the most potent carcinogens to date [2]. The permissible levels of aflatoxin B_1_ in various food items that are highly prone to contamination are stipulated by the Chinese food hygiene standards. For corn, peanuts, and peanut oil, the permitted level of aflatoxin B_1_ is set at ≤20 μg/kg [3]. The regulation of the levels of AFB_1_ in grain, peanuts, and their products has been established by the European Union and other nations. For peanuts intended for immediate consumption, the permissible level of AFB_1_ must not exceed 2 μg/kg. Additionally, for import purposes, peanuts utilized as food ingredients must not exceed an AFB_1_ content of 8 μg/kg [4].

Several techniques have been reported for the detection and analysis of AFB_1_, including high-performance liquid chromatography (HPLC) [5], enzyme-linked immunosorbent assay (ELISA) [6], and thin-layer chromatography (TLC) [7]. Although these aforementioned methods offer high sensitivity and accuracy, they also come with distinct limitations. These methods necessitate experimenters to possess proficient operational skills, as well as expensive equipment, materials, and intricate sample preparation procedures [8]. In addition to the aforementioned methods, in recent years, emerging techniques such as electrochemistry [9], fluorescence [10], chemiluminescence [11], optical fibers [12], and surface plasmon resonance (SPR) [13] have also been employed for the detection of AFB_1_ concentration.

Researchers have demonstrated significant interest in electrochemical immunosensors as a promising technology for detecting AFB_1_ in food. Their attractiveness stems from their ability to provide high sensitivity, cost-effectiveness, and rapid response times. In recent years, carbon nanomaterials have emerged as a prominent focus of research due to their exceptional characteristics of high specific surface area [14] and excellent conductivity [15]. These properties render them widely applicable in the electrochemical detection of AFB_1_, leading to significant improvements in both the accuracy and efficiency of the detection process. Shi et al. magnetically stirred a dispersion of PVP, ascorbic acid, and COOH-GO at 90 °C for 10 min. Subsequently, they mixed the dispersion with a HAuCl4 solution for 3 h. Through this procedure, they obtained Au-COOH-GO nanocomposites to fabricate electrochemical immunosensors for AFB_1_ detection [16]. Srivastava et al. synthesized graphene oxide (GO) using the modified Hummers method. Initially, graphite powder was pre-oxidized by reacting it with a mixture of H_2_SO_4_, K_2_S_2_O_8_, and P_2_O_5_ for 4 h at 80 °C. Subsequently, it was stirred in H_2_SO_4_/H_3_PO_4_, followed by the addition of KMnO4, and the mixture was stirred for 15 h at 50 °C to obtain GO. The synthesized GO was then utilized in the fabricate electrochemical immunosensors for detecting AFB_1_ [17]. Bhardwaj et al. synthesized GO also using the modified Hummers method and subsequently subjected the resulting GO to hydrothermal treatment at 200 °C for 10 h to obtain GQDs. The GQDs were synthesized to fabricate electrochemical immunosensors for the detection of AFB_1_ [18]. Although the electrochemical methods developed above demonstrate good performance in detecting AFB_1_, the fabrication process of the nanocomposites is intricate and the preparation efficiency is relatively low.

In conclusion, the application of graphene and its modified materials in electrochemical immunosensors has provided a new approach for the detection of AFB_1_. These studies have laid the foundation for the development of more sensitive and highly selective methods for detecting AFB_1_, holding significant importance in the field of food safety [19,20]. Additionally, graphene has significant advantages due to its large specific surface area and high conductivity for electrochemical biosensors. However, it is naturally hydrophobic and tends to aggregate in hydrophilic solvents [21]. Due to its excellent film-forming properties and effective dispersion effect, chitosan has emerged as a popular dispersant for graphene [22]. Furthermore, chitosan exhibits excellent biocompatibility and the ability to immobilize various functional groups, rendering it a desirable substrate for the immobilization of biosensors [23]. In this work, CS-GNs nanocomposites were synthesized and immobilized on glass carbon electrodes (GCE). Chitosan, known for its biocompatibility, was utilized to immobilize the AFB_1_ antibody, thereby enhancing the specificity of the sensor. Through this approach, a straightforward, effective, and exceptionally precise electrochemical immunosensor was constructed and utilized for the detection of AFB_1_ concentration in actual corn samples.

## 2. Results and Discussion

### 2.1. Characterization of CS-GNs Nanocomposites

**Figure 1:**
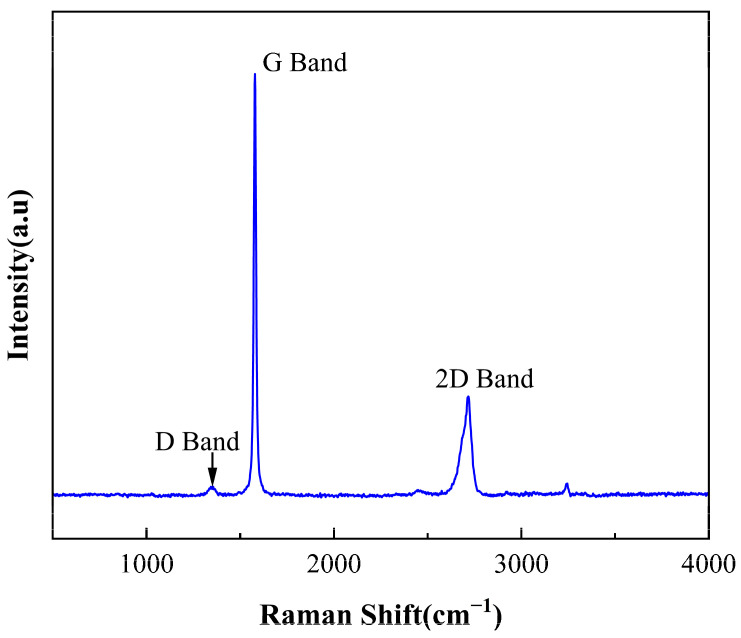
Raman spectra of GNs.

Raman spectroscopy is a powerful tool for characterizing the structure and properties of graphene [24]. We conducted an analysis of GN samples using the Raman spectroscopy technique. As shown in Figure 1, three main peaks are observed, namely the G band, 2D band, and D band. The G band is located at approximately 1579 cm⁻^1^, representing the E2g vibrational mode within the GNs lattice, corresponding to the in-plane vibrations between carbon atoms. The 2D band is located at around 2717 cm⁻^1^, representing the double resonance mode between the layers of GNs. The D band is situated at approximately 1355 cm⁻^1^, indicating structural distortions caused by defects and impurities within GNs. It is notable that the intensity of the D band is relatively weak, suggesting that the GNs sample exhibits high crystallinity and fewer defects.

**Figure 2:**
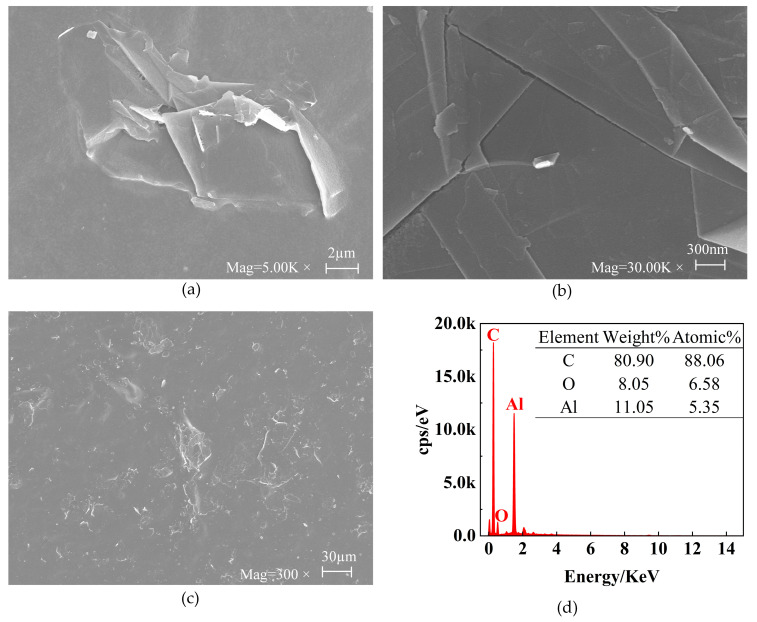
SEM images (**a**–**c**) of CS-GNs nanocomposites at different magnifications. EDS patterns (**d**) of CS-GNs nanocomposites.

The morphology of the CS-GNs was characterized using SEM and EDS. To study the SEM of CS-GNs nanocomposites, a droplet of CS-GN dispersion was applied onto tin foil for scanning. The SEM image of Figure 2a shows the size dimensions of the GNs around 10 µm. The SEM image in Figure 2b illustrates the structure of the GNs. The image clearly showed the structure of the overlapped graphene sheets, with visible layer edges and folds. The SEM image in Figure 2c shows the dispersion of GNs. It is evident that the graphene was uniformly distributed in the chitosan solution, indicating that graphene is relatively well dispersed in chitosan, with a homogeneous morphology and a substantial biocompatible membrane surface area.

Furthermore, the EDS patterns of CS-GNs nanocomposites are depicted in Figure 2d. From the patterns, it is evident that the main elements detected include C, O, and Al elements. Among them, the weight percentage of element C was 80.90%, with an atomic percentage of 88.06%; the weight percentage of the element O was 8.05% and the atomic percentage was 6.58%; and the weight percentage of element Al was 11.05% and the atomic percentage was 5.35%. Since tin foil was utilized as the substrate for the SEM inspection of CS-GNs nanocomposites, and the predominant element in tin foil paper is aluminum, a significant peak of aluminum is observed in the EDS pattern. These results showed that the CS-GNs nanocomposites were successfully prepared.

**Figure 3:**
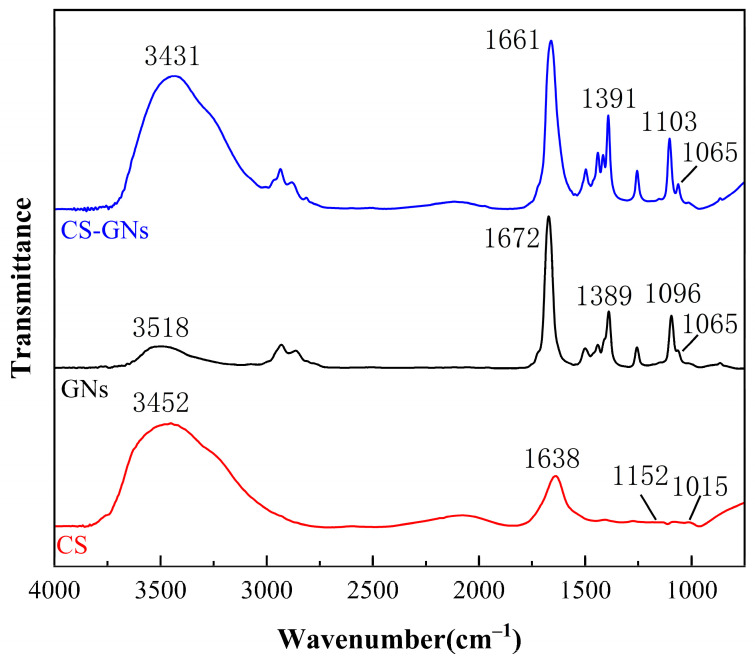
FTIR spectra of GNs, CS, and CS-GNs.

The FTIR spectra of the GN dispersion solution, CS solution, and CS-GNs nanocomposites are shown in Figure 3. It can be observed that there are more oxygen-containing functional groups in the GNs dispersion. The vibrational bands observed around 3158 cm^−1^ correspond to the −OH stretching vibration peak. The stretching vibration peak of the skeleton C=C is at 1389 cm^−1^. The stretching vibration peak at 1672 cm^−1^ corresponds to C=O, and the stretching vibration peak of the epoxy bond C–O–C is at 1095 cm^−1^. In the CS solution, the N–H stretching vibrations originating from amino and −NH_2_ groups are observed at 3452 cm^−1^. The peak at 1638 cm^−1^ corresponds to the stretching vibration of the C=O group in acetylated amino units. Additionally, the peaks observed at 1152 cm^−1^ and 1015 cm^−1^ are attributed to the stretching vibrations of the C_6_–OH primary alcohol group and the C_3_–OH secondary alcohol group in CS, respectively. The FTIR spectra of CS-GNs is generally similar to that of the dispersed GNs and the CS solution, with no new characteristic peaks observed. This indicates that there is no chemical reaction between GNs and CS. The peak observed at 3431 cm^−1^ in the CS-GNs spectrum is attributed to the interaction between the −OH groups of GNs and the −NH_2_ groups of CS. Compared to the FTIR spectra of GNs and CS, the intensities of the characteristic peaks in the CS-GNs spectrum are enhanced, indicating the formation of hydrogen bond interactions between GNs and CS.

Due to its structural characteristics, CS-GNs exhibit a relatively high specific surface area. Firstly, graphene nanosheets, as a component of CS-GNs, possess a two-dimensional structure and a monolayer arrangement of carbon atoms, resulting in a significantly large specific surface area [25]. This characteristic endows graphene nanosheets with excellent performance in adsorption, catalysis, and other fields. Secondly, chitosan is a polysaccharide polymer containing abundant hydroxyl functional groups, enabling it to interact favorably with graphene nanosheets at the molecular level [26]. Through the composite of chitosan with graphene, a greater surface area of nanocomposite materials can be achieved. The advantage of this composite structure not only increases its specific surface area but also enhances its performance in applications such as adsorption, catalysis, sensing, and others, making it a material with promising and wide-ranging application prospects [27].

### 2.2. Characterization of the Immunosensor

**Figure 4:**
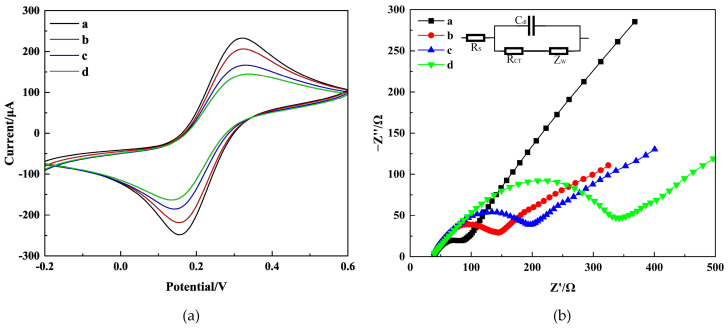
CV (**a**) and EIS (**b**) on CS-GNs/GCE (curve a), anti-AFB_1_/CS-GNs/GCE (curve b), BSA/anti-AFB_1_/CS-GNs/GCE (curve c), AFB_1_/BSA/anti-AFB_1_/CS-GNs/GCE (curve d) in 0.2 M pH = 7.2 PBS containing 5.0 mM K_3_[Fe(CN)_6_] and 0.1 M KCl. The concentration of AFB_1_ is 15 ng/mL.

To examine the characteristics of the immunosensor interface, experiments were conducted using CV and EIS methods. The base solution consisted of 0.2 M PBS containing 5.0 mM K_3_[Fe(CN)_6_] and 0.1 M KCl. Figure 4 shows the CV and EIS scan the results of different modified electrodes: bare CS-GNs/GCE (curve a), anti-AFB_1_/CS-GNs/GCE (curve b), BSA/anti-AFB_1_/CS-GNs/GCE (curve c), and AFB_1_/BSA/anti-AFB_1_/CS-GNs/GCE (curve d).

CV scans were performed on the modified electrodes at a rate of 25 mV/S between −0.2 and 0.6 V. In Figure 4a, two separate peaks are displayed by the CS-GNs/GCE with a $\Delta E_{p}$ ($E_{pa}-E_{pc}$) value of 163 mV. The current values for $I_{pa}$ and $I_{pc}$ are 232.7 µA and −248.3 µA, respectively. After the incubation of the antibodies on CS-GNs/GCE, the $\Delta E_{p}$ value exhibited an increase to 168 mV, while the $I_{pa}$ and $I_{pc}$ values displayed a decrease to 206.4 µA and −218.9 µA, respectively. Furthermore, the obstruction of active sites by antibodies caused a hindrance to the electron transfer between [Fe(CN)_6_]^3−^ and the electrode. After BSA was immobilized on the electrode surface, the peak current decreased even further. The $\Delta E_{p}$ value increased to 185 mV, and the $I_{pa}$ and $I_{pc}$ values decreased to 166.7 µA and −185.4 µA, respectively. The results indicated that the active sites responsible for nonspecific adsorption were successfully obstructed [28]. When the immunosensor was incubated with AFB_1_ (15 ng/mL), a clear reduction in peak current was detected. The $\Delta E_{p}$ value increased to 198 mV, and the $I_{pa}$ and $I_{pc}$ values decreased to 144.7 µA and −163.4 µA, respectively. The results indicated that the immunoreaction occurred, and the AFB_1_ captured on the electrode surface hindered the reaction of [Fe(CN)_6_]^3−^, indicating the successful formation of the immune complex on the electrode surface [29].

EIS was also a powerful tool for characterizing the step-by-step manufacturing process of the electrode [30]. The electron transfer resistance ($R_{CT}$) was analyzed by fitting the diameter of the semicircle using the Randles equivalent circuit (inset in Figure 4b). The Nyquist plot presents the $R_{CT}$ behavior of an electrode. A semicircle diameter forms at the higher frequency region, indicating an electron-limiting process. Meanwhile, the low frequency region exhibits a diffusion-controlled process [31]. As depicted in Figure 4b, the diameter of the semicircle observed in the CS-GNs/GCE was significantly smaller compared to the others, indicating a large electrode surface area and superior conductivity. The $R_{CT}$ value of CS-GNs/GCE was measured at 66.72 Ω. Following the incubation of antibodies on the CS-GNs/GCE, the $R_{CT}$ value increased to 129.2 Ω, surpassing that of the CS-GNs/GCE. This may be attributed to the blocking of electron transfer by the antibodies. After BSA was immobilized on the electrode surface, the semicircular domain increased, and the $R_{CT}$ value was found to increase up to 197.2 Ω. This indicates that the active sites causing nonspecific adsorption were successfully blocked by BSA. Finally, the AFB_1_ (15 ng/mL) was immobilized on the electrode, a significant increase in the semicircular domain was observed, and the $R_{CT}$ value increased to 320.1 Ω. This result suggested that the immune complex effectively formed on the electrode surface, thereby impeding the electron transfer. As expected, EIS was utilized to assess the precise state of the immunosensor during each stage of assembly. The results indicate that the immunosensor was successfully fabricated. Consequently, data derived from both CV and EIS showed that the successful fabrication of the immunosensor.

### 2.3. Optimization of Experimental Conditions

To examine the immunosensor’s optimum sensing capabilities, we investigated the impacts of various factors on its performance. These factors included the concentration of the immobilized antibody, pH levels, incubation duration of the antibody, and immunoreaction time.

**Figure 5:**
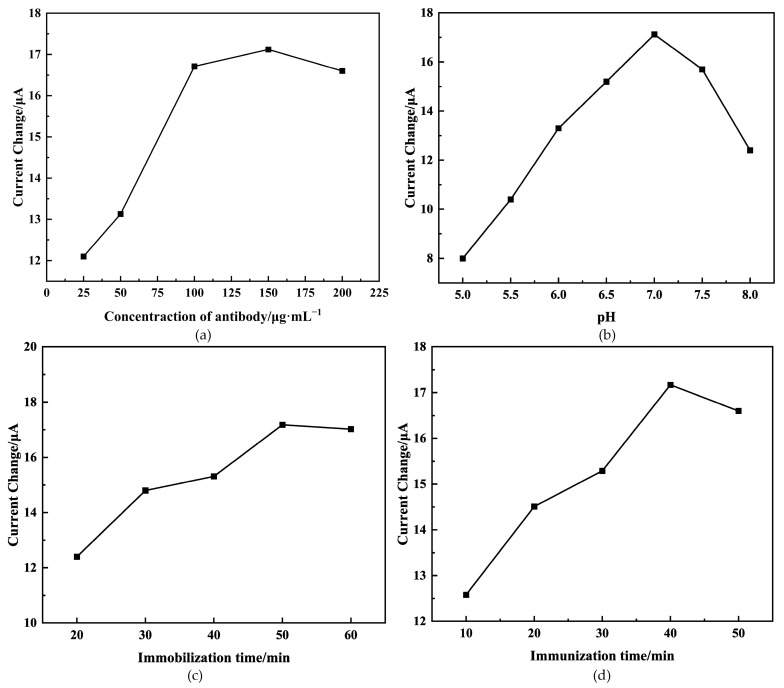
Effects of antibody concentration (**a**), pH (**b**), antibody immobilization time (**c**), and immunoreaction time (**d**) on peak current change of immunosensor. The concentration of antigen is 15 ng/mL.

The performance of the sensor is highly dependent on the concentration of antibodies immobilized on the electrode surface, as they create binding sites for antigens. We conducted an experiment to investigate the impact of different antibody concentrations (25, 50, 100, 150, and 200 µg/mL) on detecting AFB_1_ at a concentration of 15 ng/mL using the immunosensor. Figure 5a illustrates the change in peak current (ΔI) before and after the immunoreaction. It is observed that ΔI increases until reaching 150 μg/mL, after which it begins to decrease. This may be attributed to antibodies’ saturation at this concentration, consistent with the findings in the existing literature [32]. Consequently, 150 μg/mL was determined as the optimal antibody concentration.

The pH value of the base solution was a crucial parameter, potentially resulting in protein denaturalization or the instability of the immunosensor [33]. Figure 5b illustrates that the peak current change (ΔI) gradually increased with the pH value of the base solution increased, reaching its peak at 7.0. This happens because extreme acidity or alkalinity can damage the immobilized protein, especially under alkaline conditions [34]. As a result, the pH value of the base solution was adjusted to 7.0 for further investigation.

The performance of the sensor can be affected by the duration of antibody immobilization. As depicted in Figure 5c, the change in peak current (ΔI) exhibited a gradual increase with prolonged antibody immobilization time until it reached a plateau at 50 min. This could be attributed to the antibody reaching its saturation point in terms of activity [35]. Therefore, based on the experiment, 50 min was determined as the optimal duration.

The duration of the immunoreaction between the antigen and antibody significantly affects the performance of the sensor. As depicted in Figure 5d, there was a gradual increase in the peak current change (ΔI), increasing the immunoreaction time, which eventually leveled off at 40 min. This outcome suggests that the immunoreaction between the antigen and antibody reached saturation after 40 min. Thus, 40 min was identified as the optimal duration for the immunoreaction between the antigen and antibody.

### 2.4. Analytical Performance

Under the optimal conditions, the performance of the prepared immunosensor was evaluated for various concentrations of AFB_1_ using the DPV technique.

**Figure 6:**
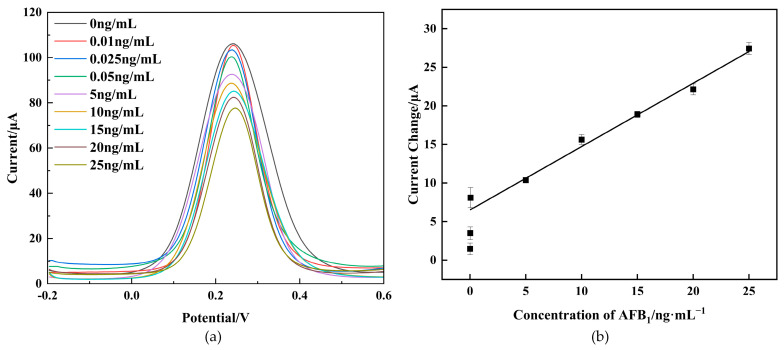
Immunosensor response to DPV at different concentrations of AFB_1_ (**a**). Δ*I* calibration curves of the immunosensor for different concentrations of AFB_1_ (**b**).

As depicted in Figure 6a, the DPV peak currents exhibited a notable decrease with an increasing AFB_1_ concentration within the range of 0–25 ng/mL. This decrease can be attributed to the heightened hindrance of the immunocomplex to electron transfer. As depicted in Figure 6b, the current change (ΔI) before and after immunization displayed a linear relationship with AFB_1_ concentrations from 0.05 ng/mL to 25 ng/mL, with a low detection limit of 0.021 ng/mL (S/N=3). The limit of detection (LOD) was determined using the regression curve parameters, $LOD=3\cdot S_{b}/s$, where “$S_{b}$” represents the ard deviation of the blank sample and “s” represents the slope [36].The calibrated regression equation is:

ΔI=0.822·C+6.504,

with a correlation coefficient of 0.99. The proposed immunosensor was compared with other reported AFB_1_ immunosensors reported in the literature. The acceptable linear range and detection limit of the proposed immunosensor are described in Table 1, indicating its excellent performance for AFB_1_ detection. The outstanding electrochemical performance of the proposed immunosensor stemmed from the large surface area and exceptional conductivity of CS-GNs nanocomposites.

The LOD of the developed immunosensor surpassed that of most reported electrochemical methods for the detection of AFB_1_. The LOD of the immunosensor was comparable to that achieved by Li et al. [37], which developed a biosensor based on aptamers for AFB_1_ detection. However, the biosensor developed by Li et al. [37] had a complex structure, was costly, and cumbersome to prepare.

### 2.5. Reproducibility, Stability, and Selectivity

**Figure 7:**
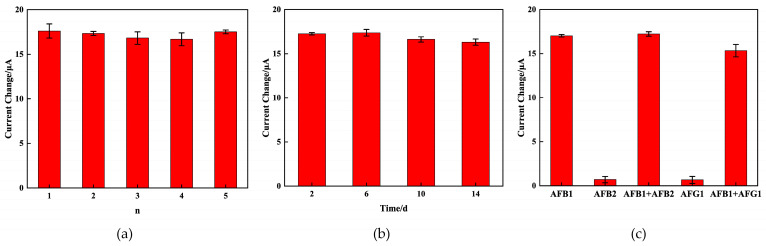
Amperometric change response of the immunosensor to 5 different electrodes treated in the same way (**a**); the time stability study of the immunosensor (**b**); and the current change responses of the immunosensor to AFB_1_, AFB_2_, AFB_1_ + AFB_2_, AFG_1_, AFB_1_ + AFG_1_ (**c**).

In order to investigate the reproducibility of the immunosensor, five electrodes were tested to detect 15 ng/mL AFB_1_ under the same conditions. The results are depicted in Figure 7a, the relative standard deviation (RSD) of the AFB_1_ measurements for the five sensors is 2.4%, which proved that the proposed immunosensor has excellent reproducibility.

The stability of the immunosensor was assessed by detecting the electrochemical response after the immunosensors were stored at 4 °C for 2, 6, 10, and 14 days. As depicted in Figure 7b, after 14 days of storage, the electrochemical response retained 94.42% of the initial current for 5 ng/mL AFB_1_, which indicated the significant stability of the immunosensor.

The specificity of the prepared immunosensor was also crucial for assessing its performance. The specificity was evaluated using interfering substances, which consisted of 15 ng/mL of AFB_2_ and AFG_1_. As observed in Figure 7c, the peak current change (ΔI) before and after immunization with pure interfering substances exhibited no noticeable variation. The observed peak current change (ΔI) before and after immunization with the mixture solution showed similarity to that of the 15 ng/mL AFB_1_ standard solution. All the above observations demonstrate that the immunosensor exhibited a commendable level of specificity.

### 2.6. Detection of AFB_1_ in Corn Samples

In order to assess the precision of the immunosensor, spiked recoveries were measured in pretreated samples of corn. The standard addition method was employed to assess the application of the proposed immunosensor in corn samples. AFB1 was added to the corn samples at spiked concentrations of 5 ng/mL, 10 ng/mL, and 15 ng/mL, respectively. As shown in Table 2, the range of the recovery was from 97.3 to 101.4%. These results demonstrated the practicality of the immunosensor in effectively analyzing the target AFB1 concentrations in real samples.

## 3. Materials and Methods

### 3.1. Materials and Apparatus

The graphene nanosheets (2 nm, with a diameter of 2~3 µm) were purchased from Nanjing Xianfeng Nanomaterials Technology Co., Ltd., located in Nanjing, China. Chitosan was obtained from China National Pharmaceutical Group Chemical Reagent Co., Ltd. (Shanghai, China). Aflatoxin B_1_ (AFB_1_), aflatoxin B_2_ (AFB_2_), and aflatoxin G_1_ (AFG_1_) standard solution (in acetonitrile, 10 µg/mL) were sourced from Beijing ZhongkeErhuan Technology Co., Ltd. (Beijing, China). The anti-Aflatoxin B_1_ antibody was provided by Shanghai Sangong Biological Engineering Co., Ltd. (Shanghai, China). Bovine serum albumin (BSA) and 1-ethyl-(3-dimethylaminopropyl) carbodiimide hydrochloride (EDC.HCl) were obtained from Hefei Genial Biotech Co., Ltd. (Hefei, China). *N*-hydroxysuccinimide (NHS), *N*,*N*-dimethylformamide (DMF), and phosphate-buffered saline (PBS) at a pH range of 7.2–7.4 were procured from Shanghai Titan Technology Co., Ltd. (Shanghai, China), for experimental use. P-aminobenzoic acid (PABA) was acquired from Hefei Qiansheng Biological Technology Co., Ltd. (Hefei, China).

The electrochemical characterization tests, including differential pulse voltammetry (DPV), cyclic voltammetry (CV), and electrochemical impedance spectroscopy (EIS), were performed using the CHI-760E electrochemical workstation from Shanghai Chenhua Instrument Co., Ltd. (Shanghai, China). The electrochemical experiment utilized a conventional three-electrode system, comprising a saturated KCl Ag/AgCl electrode as the reference electrode, a platinum wire (Pt) electrode as the counter electrode, and a glass carbon electrode (GCE) as the working electrode. The scanning electron microscope (SEM) images were captured using the Sigma 300 hot-field scanning electron microscope (Carl Zeiss, Oberkochen, Germany).

### 3.2. Methods

#### 3.2.1. Preparation of CS-GNs Nanocomposites

The powder of 5 mg chitosan was dissolved in 5 mL of 1.0% (*v*/*v*) acetic acid and stirred with a magnetic stirrer for 1 h. After complete dispersion, the solution was stored at 4 °C for later use. Then, 5 mg of graphene nanosheets was dissolved in 5 mL of anhydrous ethanol and sonicated for 10 h. The supernatant was discarded after centrifugation in a centrifuge at 9000 rpm for 15 min. Then, 5 mL of DMF was added and sonicated for more than 2 h until completely dispersed. Subsequently, 5 mL of the prepared graphene dispersion was taken and mixed with 5 mL of prepared CS solution, followed by ultrasonication for 2 h to obtain a uniform CS-GNs dispersion. The CS-GNs dispersion was stored at 4 °C for further use.

#### 3.2.2. Fabrication of the Immunosensor

Before modification, the GCE was treated by a typical purification method. Initially, the bare GCE was polished with a polishing powder containing 0.05 μm Al_2_O_3_ particles until achieving a highly reflective, mirror-like surface. Secondly, the electrode was cleaned 5 min in ethanol and distilled water, and then dried at room temperature. To activate the GCE, the dried electrode was subjected to cyclic voltammetry scanning (−0.3–1.5 V, 50 mV/s) in a 0.5 M H_2_SO_4_ solution for 15 cycles. Subsequently, the GCE was cleaned by ultrasonication in distilled water for 10 min and rinsed thoroughly with abundant distilled water.

After being dried at room temperature, the cleaned GCE was subjected to CV scanning (−1.5~1.0 V, 50 mV/s) in 5 mM p-aminobenzoic acid (PABA) solution for 15 cycles, followed by rinsing with distilled water and dried at room temperature. Finally, 10 µL of CS-GNs nanocomposites dispersion was carefully dropped onto the surface of the GCE.

Before immobilizing the antibodies, the surface of CS-GNs/GCE was initially activated using EDC: NHS coupling chemistry. Here, EDC (0.4 M) served as a coupling agent, while NHS (0.1 M) acted as an activator for the covalent immobilization of biomolecules [41].

**Figure 8:**
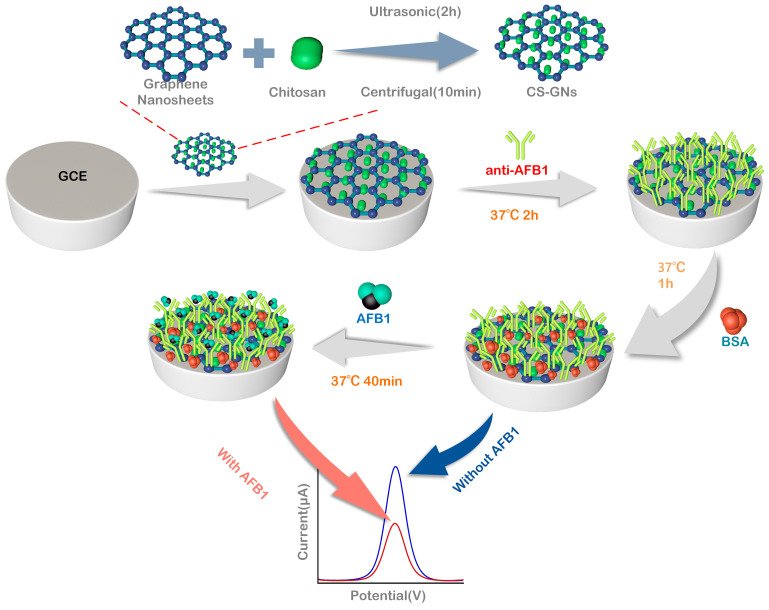
Schematic diagrams for the preparation of CS-GN nanohybrids and the electrochemical immunosensor.

After the activation, CS-GNs/GCE was thoroughly washed with PBS. Subsequently, 10 µL of 150 µg/mL anti-AFB_1_ was carefully dropped onto the surface of the CS-GNs/GCE and then incubated at 37 °C for 50 min. Following that, the fabricated electrode was rinsed with PBS to remove the physically adsorbed antibodies. Subsequently, the electrode was incubated in 10 µL 3% BSA solution at 37 °C for 1 h, in order to block any unreacted active sites on the surface. Afterwards, the electrode was thoroughly rinsed with PBS once more, resulting in the successful fabrication of the AFB1 electrochemical immunosensor using CS-GNs/GCE, which was then stored at 4 °C for further use. Thereafter, the electrode was dropped with 10 μL of AFB_1_ solution with diverse concentrations and incubated at 37 °C for 40 min. The physically adsorbed AFB_1_ antigen molecules were washed away by PBS, and then electrochemically tested by the DPV method in the base solution (5 mM K_3_[Fe(CN)_6_] + 0.1 M KCl + 0.2 M PBS). The DPV peak current change (Δ*I*) before and after the immunoreaction served as the basis for quantifying the AFB_1_ concentration in the samples. Figure 8 shows the preparation of CS-GNs nanocomposites and outlines the process for preparing the electrochemical immunosensor.

#### 3.2.3. Preparation of Spiked Samples

The spiked sample was prepared using a previously established method [42]. The unaffected corn samples were crushed and ground into powder, weighed 30 mg in a centrifuge tube, added to 30 mL of acetonitrile/water (8:2 *v*/*v*) solution, ultrasonicated for 4 h until completely dispersed, and then centrifuged at 9000 r/min for 15 min. The resulting supernatant collected and diluted multiple concentrations with PBS, and the samples were subsequently spiked with varying concentrations of AFB_1_ (5.0, 10.0, 15.0 ng/mL), before being stored at 4 °C until use.

## 4. Conclusions

In this work, we designed an electrochemical immunosensor based on chitosan graphene nanosheets (CS-GNs) for the detection of AFB_1_ concentration in corn samples. The CS-GNs nanocomposites exhibited a large specific surface area, excellent biocompatibility, and high electrochemical activity. These properties facilitate the immobilization of antibodies and enhance the rate of electron transfer. The obtained CS-GNs nanocomposites were surface characterized using SEM. The optimization of antibody concentration, pH, antibody incubation time, and immunoreaction time was based on the DPV method. With the best conditions, the change in DPV peak currents before and after immunization was linear over the concentration range of 0.05–25 ng/mL AFB_1_, with a detection limit of 0.021 ng/mL (S/N=3). The developed immunosensor exhibited favorable reproducibility, stability, and specificity for detecting the concentration of AFB_1_ in corn samples. Additionally, the recovery of AFB_1_ detection in corn samples ranged from 97.3% to 101.4%. The results show that the developed AFB_1_ immunosensor possesses the advantages of simplicity, sensitivity, and high selectivity. This makes it a valuable reference for the detection and analysis of other biomolecules.

## Figures and Tables

**Table 1:** Comparison of the proposed immunosensor and other sensors.

Immunosensors	Linear Range (ng/mL)	Detection Limit (ng/mL)	References
AFB_1_/Fc-apt/MCH/cDNA/AuNPs/THI-rGO/GCE	0.05–20	0.016	[37]
AFB_1_/BSA/anti-AFB_1_/AuNPs/Zn/Ni-ZIF-8-800@Graphene/GCE	0.18–100	0.18	[38]
AFB_1_/BSA/anti-AFB_1_/Au@PEI@CNFs/GCE	0.05–25	0.027	[39]
AFB_1_/BSA/anti-AFB_1_/Au-COOH-GO/GCE	0.05–25	0.05	[16]
AFB_1_/MCH/pept/porous/AuNPs/GCE	10–20,000	0.94	[40]
AFB_1_/BSA/anti-AFB_1_/CS-GNs/GCE	0.05–25	0.021	This work

**Table 2:** Recovery for different concentrations of AFB_1_ spiked in corn samples.

Samples	Added AFB_1_ (ng/mL)	Found AFB_1_ (ng/mL)	Recovery (%)
1	5.00	4.86	97.3
2	10.00	10.14	101.4
3	15.00	14.64	97.6

## Data Availability

Data are contained within the article.
